# Lamivudine, a reverse transcriptase inhibitor, rescues cognitive deficits in a mouse model of down syndrome

**DOI:** 10.1111/jcmm.17411

**Published:** 2022-06-28

**Authors:** Maria Martinez de Lagran, Aleix Elizalde‐Torrent, Roger Paredes, Bonaventura Clotet, Mara Dierssen

**Affiliations:** ^1^ Centre for Genomic Regulation (CRG) The Barcelona Institute of Science and Technology Barcelona Spain; ^2^ IrsiCaixa AIDS Research Institute Badalona Spain; ^3^ University of Vic‐Central University of Catalonia (UVic‐UCC) Vic Spain; ^4^ Universitat Pompeu Fabra (UPF) Barcelona Spain; ^5^ Centro de Investigación Biomédica en Red de Enfermedades Raras (CIBERER) Barcelona Spain

**Keywords:** down syndrome, lamivudine, memory, retrotransposons, reverse transcriptase inhibitor

## Abstract

An elevated activity of retrotransposons is increasingly recognized to be implicated in a wide range of neurodegenerative and neurodevelopmental diseases. Down syndrome (DS) is the most common genetic disorder associated with intellectual disability and a genetic form of Alzheimer's disease. For this reason, we hypothesized that treatment with reverse transcriptase inhibitors could ameliorate DS phenotypes. In this proof of concept study, we treated trisomic (Ts65Dn) mice, a model of DS, with lamivudine, a reverse transcriptase inhibitor. We detected a significant improvement of neurobehavioural phenotypes, and a complete rescue of the hippocampal‐dependent recognition memory upon treatment with lamivudine. Despite clinical studies in patients with DS are warranted, this study lays the groundwork for a novel and actionable therapeutic approach.

## 1 INTRODUCTION

Down syndrome (DS) is the most common known genetic disorder associated with moderate to severe intellectual disability due to a total or partial trisomy of the autosomal chromosome 21 (HSA21) and a genetic form of Alzheimer's disease (AD).^1^ Overexpression of HSA21 gene products such as DYRK1A^2^, SOD1^3^ or S100B^4^ has been proposed to contribute to the neurological and neurobehavioural DS phenotypes. However, treatments targeted to candidate genes^5^ or mechanisms have only been partially successful.^6^ This is possibly explained because besides the HSA21 gene‐dosage effects, triplication of HSA21 also leads to a genome‐wide transcriptional deregulation.^7^

Recently, single‐nucleus long‐read RNA sequencing has revealed thousands of unannotated RNA transcripts containing intra‐exonic junctions, involving a myriad of genes, including the amyloid precursor protein (APP).^8^ Notably, increased brain transcription and increased copy numbers of *APP* have been linked to *APP* somatic gene recombination associated with sporadic AD and with DS, and could contribute to their cognitive deficits.^9^ Retrotransposable elements like the long interspersed nuclear element 1 (LINE1 or L1 – 6 kb) are thought to participate in this process in mammals.^10^ Increased retrotransposition is observed in cell senescence and with ageing^11^ and has been implicated in several neurodegenerative diseases including AD, frontotemporal dementia, prion disease, but also in developmental disorders such as Rett's syndrome, autism or fragile X syndrome.^12^

Nucleoside reverse transcriptase inhibitors (NRTIs) have been used to suppress retrotransposition, and thus NRTIs could theoretically improve these pathologies. Importantly, in patients with HIV‐1, NRTIs treatment is associated with reduced risk of HIV‐associated neurocognitive disorder, AD, Parkinson's disease and other dementias.^13^ Preclinical studies have shown that the reverse transcriptase inhibitor lamivudine [(−)‐L‐2′,3′‐dideoxy‐3′‐thiacytidine (3TC)] improves cognition in senescence‐accelerated prone 8 (SAMP8) mice, a model for studying human ageing and age‐related diseases^14^ and treatment with NRTIs doubled the lifespan of progeroid Sirt6‐deficient mice^15^, supporting this possibility. Last year, a clinical trial to investigate the safety and feasibility of antiretroviral therapy with lamivudine for Alzheimer's disease was launched [ClinicalTrials.gov Identifier: NCT04552795]. Although a contribution of retrotransposition to developmental disorders has also been suggested and despite the close relationship of DS with AD^16^, NRTIs have not been explored in DS. In this proof of concept study, we show a clear rescuing effect of lamivudine on hippocampal‐dependent recognition memory in Ts65Dn mice, a DS mouse model.

## 2 MATERIALS AND METHODS

Trisomic (Ts65Dn, TS) male mice were obtained by breeding B6EiC4Sn.BLiA‐Ts(1716)65Dn/DnJ females with C57BL/6 × 6JOlaHsd (B6C3F1/OlaHsd) hybrid males. Mice were genotyped, and ∼25% of the offspring presented with trisomy. Wild‐type (WT) littermates were used as controls. After weaning, male mice were group housed with three to four animals per cage on a conventional 12:12 light cycle in controlled environmental conditions of humidity (50%–70%) and temperature (21 ± 1°C). Water and standard rodent chow were available ad lib. Experiments were conducted using 3–4 month‐old male mice. TS and WT mice were assigned using a simple randomization to either control conditions or lamivudine 3 mg/kg (Epivir, 10 mg/ml, oral solution) in the drinking water. 15 TS and 20 WT mice were treated with lamivudine and 14 TS and 18 WT mice were used as a control group and received water. The treatment lasted 4 months. Behavioural testing was performed before the treatment and 1 and 4 months after starting the treatment. Behavioural experiments were conducted during the light phase of the light/dark cycle between 10 am and 2 pm, and performed by trained observers blind to genotype. The novel object recognition test and plus maze were videotaped using a computer‐assisted data acquisition system (SMART, Panlab Harvard Apparatus). Less stressful tests were performed first and each behavioural test was separated by at least 24 h. The order of the tests was as follows: locomotor activity, novel object recognition and elevated plus maze. All procedures had authorization from the Barcelona Biomedical Research Park Animal Research Ethics Committee (PRBB‐CEEA) and the local government (Generalitat de Catalunya) and were conducted according to the European Directive 2010/63/EU and Spanish regulations RD 53/2013.

### 2.1 Locomotor activity

Spontaneous locomotor activity in the home cage was measured using an automated infrared light‐beam monitoring system (ActiMot2, TSE system) and the ambulatory beam breaks (X axis) were counted for 24 h. Total general activity was analysed. Mice were isolated for assessing locomotor activity during 24 h and immediately regrouped after the test.

### 2.2 Novel object recognition test

The apparatus consisted of a rectangular open‐field arena (50 cm long × 50 cm wide × 50 cm high) made of black Plexiglas. Animals' behaviour was monitored using System Motor Activity Record and Tracking software (SMART, Panlab Harvard Apparatus). On the first day, mice were habituated to the arena for 5 min. On the second day, mice were presented with two identical objects, for 10 min. Subjects failing to complete a minimum of 20 s of exploration during the familiarization session were excluded for posterior analysis. Twenty‐four hours after (test session), mice were presented with one familiar object and one novel one, for 5 min. The discrimination index was calculated as ([time exploring the novel object – time exploring the familiar object]/total time of exploration) * 100 [48]. Exploratory behaviour was defined as the animal directing its nose towards the object at a distance of <2 cm and manually registered by the experimenter. Sitting on or resting against the object was not considered as exploration. All the objects used were made of plastic and induced similar exploration levels. The arena and objects were deeply cleaned between animals to avoid olfactory cues.

### 2.3 Elevated plus maze

The elevated plus maze paradigm was used to study anxiety‐related behaviour. The apparatus consists of a cross‐shaped platform (four arms faced two to two with a length of 40 cm and 8 cm width) elevated 40 cm from the floor. Two opposing arms are protected by black walls and the other two are left unprotected. Because of the distance to the ground, the open arm is an aversive environment for the mouse, so it tends to remain in the closed arms. The proportion of time spent in open versus closed arms during 5 min is considered a measure of anxiety. Distance travelled was analysed as a measure of the activity in the apparatus.

### 2.4 Statistical analyses

Two‐way analysis of variance (anova) repeated measures was used for testing genotype and treatment differences on the locomotor activity and the elevated plus maze. Two‐way anova was used to analyse the novel object recognition test. Tukey or Bonferroni post hoc tests were used as a correction between pairwise comparisons. All statistical analyses were performed using Statistical Package for the Social Sciences (SPSS) software (version 19.0) and GraphPad Prism (version 8.01).

## 3 RESULTS

### 3.1 Lamivudine ameliorated hyperactivity in TS mice

**FIGURE 1:**
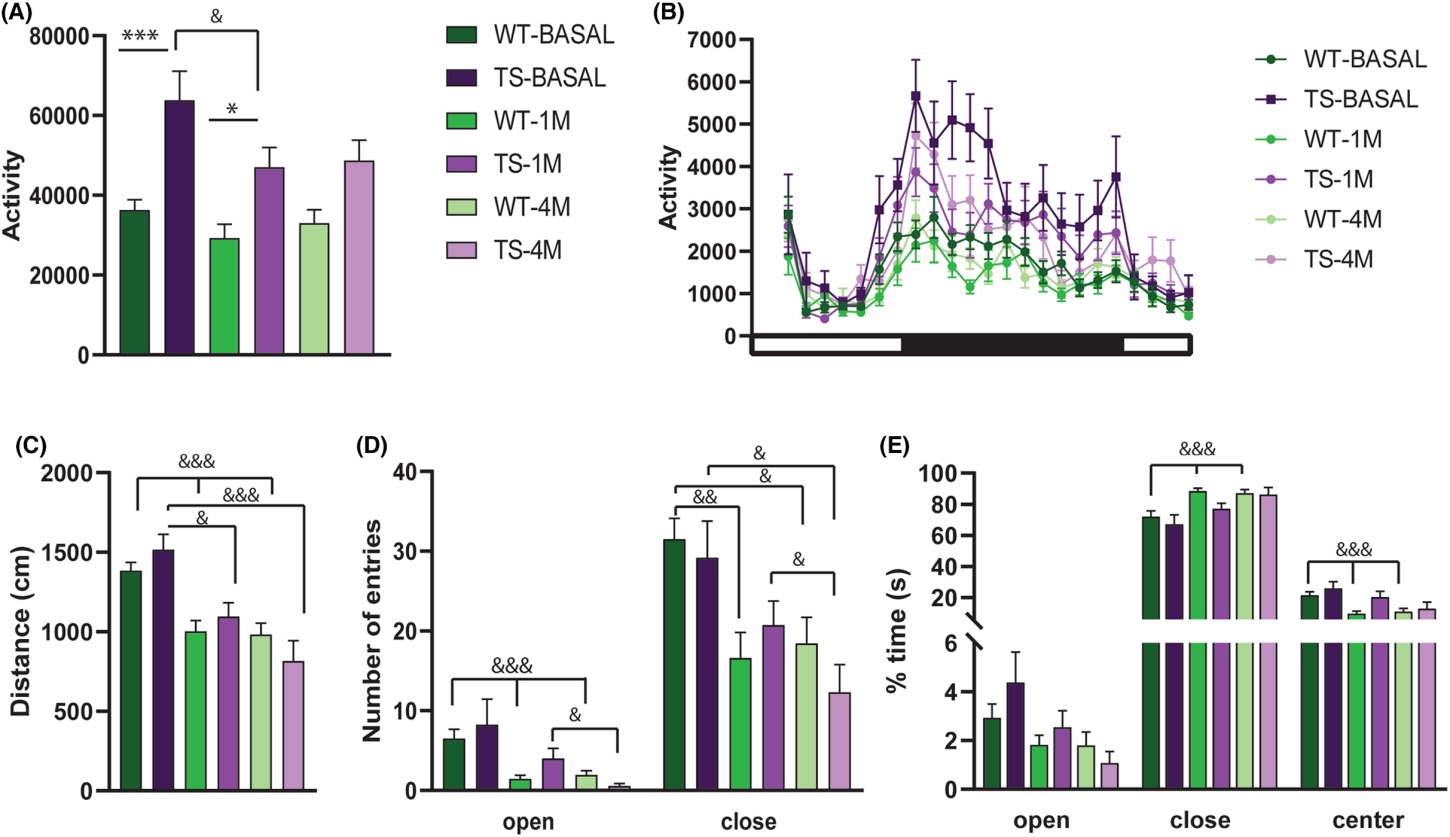
Locomotor activity and anxiety‐like behaviour in TS and respective WT in basal conditions and after 1 or 4 months of treatment with lamivudine. The upper panel depicts (A) total activity and (B) circadian activity for 24 h measured as ambulatory (X axis) beam breaks. In (B) the dark square under the X‐axis represents the dark phase of the cycle (WT *n* = 20, TS *n* = 15). Anxiety‐like behaviour was assessed in elevated plus maze. The lower panel depicts the (C) total distance travelled, (D) number of entries and (E) percentage of time spent in each zone of the elevated plus maze (WT *n* = 20, TS *n* = 12). Data are represented as mean ± SEM. Genotype effect *p < 0.05, **p < 0.01, ***p < 0.001, Treatment effect ^&^ *p* < 0.05, ^&&^ *p* < 0.01; ^&&&^ *p* < 0.001

We analysed locomotor activity of WT and TS mice in baseline conditions and the effect of lamivudine in both genotypes one and 4 months after starting the treatment. Statistical analysis showed a significant genotype effect (F[1,22] = 15.76 *p* < 0.01). In baseline conditions, TS mice showed significantly increased total locomotor activity as compared to WT mice (Bonferroni post‐hoc, *p* < 0.001, Figure 1A), as shown by an increased distance travelled during 24 h.

After 1 month of treatment with lamivudine, total locomotor activity was reduced in TS mice with respect to baseline levels, although not completely rescued to WT levels (Bonferroni post‐hoc, *p* < 0.05, Figure 1A), and the same tendency was observed after 4 months of treatment, although no statistically significant differences were observed between both genotypes (Bonferroni post‐hoc, N.S., Figure 1A). A significant effect of lamivudine (treatment effect [F2, 60] = 8.07, *p* < 0.001) was observed. However, the distance travelled was significantly reduced after one month of treatment only in TS mice (Tukey post‐hoc, TS basal vs TS‐1 M; *p* < 0.05).

These results indicate an amelioration of hyperactivity in Ts65Dn after treatment with lamivudine. When analysing the circadian pattern of activity (Figure 1B), we observed that the treatment‐associated changes of locomotor activity are mainly detected during the active (dark) phase of the circadian cycle. The increase in locomotor activity in TS mice during the dark phase are significant in basal conditions (F[1,33] = 13.53, *p* < 0.001), and are significantly reduced with the treatment (1 month of treatment: F(1,33) = 9.09, *p* < 0.01 and 4 month of treatment F(1,33) = 5.88, *p* < 0.05).

### 3.2 Lamivudine increased anxiety‐like behaviour in WT and TS mice

We also assessed anxiety‐like behaviour in the elevated plus maze paradigm to understand whether TS mice would be more sensitive to this side effect of lamivudine (Figure 1C–E). We first analysed locomotor activity (distance travelled) (Figure 1C). We did not detect genotype‐dependent differences, but both genotypes travelled a reduced distance in the maze after treatment (treatment effect F[2, 51] = 34.67, *p* < 0.001. Tukey's post‐hoc test, WT BASAL vs. WT1M *p* < 0.001, WTBASAL vs. WT4M *p* < 0.001, TSBASAL vs. TS1M *p* < 0.05, TS BASAL vs. TS4M *p* < 0.001, Figure 1C). Possibly related to this, although no significant differences between genotypes were detected in the number of entries in open or closed arms, upon treatment we detected a reduction of entries in both open (treatment effect, F[1, 38] = 15.10, *p* < 0.001, Figure 1D) and closed arms (F[2, 52] = 12.55, *p* < 0.001). The reduction in the number of entries was maintained along the treatment at 1 and 4 months both in open (Tukey's post‐hoc test, WT‐BASAL vs. WT‐1 M *p* < 0.001; WT‐BASAL vs. WT‐4 M *p* < 0.001; TS‐1 M vs. TS‐4 M *p* < 0.05) and in closed arms (Tukey's post‐hoc test, WT‐BASAL vs. WT‐1 M *p* < 0.01; WT‐BASAL vs. WT‐4 M *p* < 0.05; TS‐BASAL vs. TS‐4 M *p* < 0.05, TS‐1 M vs. TS‐4 M *p* < 0.05). As both the entries in open and closed arm were reduced, this global reduction in activity as measured also by the distance travelled may reflect the habituation to the apparatus after repeated exposures.

Anxiety‐like behaviour is expressed as an increased time spent in the close arms with respect to open arms. No differences between genotypes in the number of entries nor in the time spent in neither open nor closed were observed, indicating similar anxiety‐related behaviour in TS and WT mice. However, we detected a treatment effect in the time spent in closed arms (treatment effect, F[1.5, 46] = 15.18, *p* < 0.001, Figure 1E), that was slightly increased in both genotypes and in the centre (treatment effect, F[1.8, 55] = 11.82, *p* < 0.001), in which permanence was slightly reduced. Upon lamivudine treatment, WT mice spent significantly more time in closed arms (Tukey's post‐hoc test, WT‐BASAL vs WT‐1 M *p* < 0.001; WT‐BASAL vs WT‐4 M *p* < 0.001) and less time in open arms (non‐significant) and in the centre of the maze (Tukey's post‐hoc test, WT‐BASAL vs. WT‐1 M *p* < 0.001; WT‐BASAL vs. WT‐4 M *p* < 0.001) as compared to basal conditions, suggesting anxiety‐like effects. In the case of the TS, although the tendency is similar, no significant differences were observed.

### 3.3 Lamivudine rescues recognition memory deficits in TS mice

**FIGURE 2:**
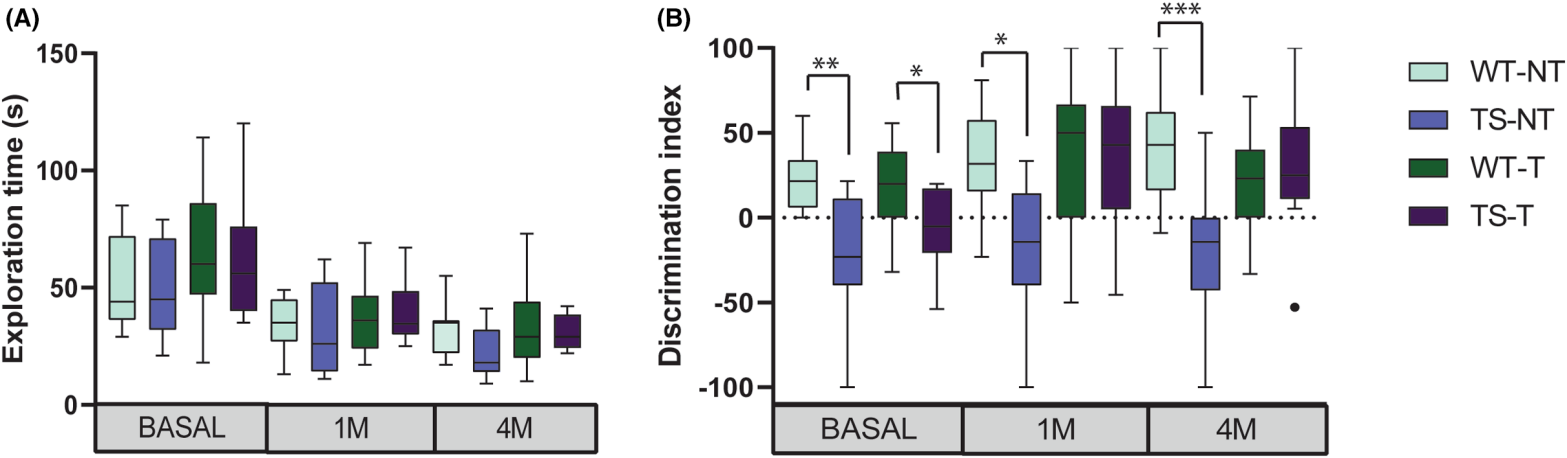
Cognitive assessment in novel object recognition test in TS and respective WT in basal conditions and after 1 or 4 months of treatment with lamivudine. Boxplots represent (A) total time of exploration during the familiarization phase and (B) the discrimination index 24 h after familiarization (treated: Wild type (WT‐T) *n* = 17, Ts65Dn (TS‐T) *n* = 13; non‐treated: Wild type (WT‐NT) *n* = 9, Ts65Dn (TS‐NT) *n* = 9). Boxplots extend from the 25th to 75th percentiles and the median is represented as a line in the box. The whiskers correspond to the maximum and minimum value excluding outliers. *p < 0.05, **p < 0.01, ***p < 0.001

Recognition memory was evaluated using the novel object recognition paradigm that has been shown to be a robust and reproducible test for studying the Ts65Dn strain^17^. For this test, we included an independent group of WT and TS receiving water in basal conditions and after 1 or 4 months to discard possible carry‐over effects of the test, and track the possible age‐related differences in recognition memory. During the familiarization test, no differences in the time of exploration were observed among the groups at baseline, 1 month or 4 months (Figure 2A). In basal conditions, TS mice showed recognition memory impairment, as shown by the significantly reduced discrimination index (Two‐way anova, F[3,47] = 1.28 *p* < 0.001, Tukey's post‐hoc test: WT‐NT vs. TS‐NT *p* < 0.01 WT‐T vs. TS‐T *p* < 0.05). One month after starting non‐treated mice continued showing cognitive impairment (F[3,47] = 5.27 *p* < 0.001, Tukey's post‐hoc test: WT‐NT vs TS‐NT *p* < 0.05), while TS treated with lamivudine, showed a complete rescue of the cognitive deficit reaching WT performance levels (Tukey's post‐hoc test: WT‐T vs. TS‐T *p* = 0.98, Figure 2B). The cognitive rescue in TS mice was maintained after 4 months of treatment (Two‐way anova, F[3,47] = 6.28 *p* < 0.001, Tukey's post‐hoc test: WT‐T vs. TS‐T *p* = 0.82, Figure 2B), whereas non‐treated Ts65Dn mice showed an even worse discrimination index (Tukey's post‐hoc test: WT‐NT vs. TS‐NT *p* < 0.001, Figure 2B), probably due to age‐associated cognitive decline.

## 4 DISCUSSION

Increased retrotransposition is implicated in neurodevelopmental diseases, so in this proof of concept study, we tested if lamivudine, a reverse transcriptase inhibitor, could reverse the cognitive impairment in a DS mouse model, the Ts65Dn (TS) mice, the most extensively validated model of DS. TS mice present hyperactivity and hippocampal‐dependent learning deficits^18^, and recapitulate several of the DS neurobehavioural and cognitive phenotypes. Lamivudine significantly improved neurobehavioural phenotypes and completely rescued hippocampal‐dependent recognition memory in young adult TS mice. It also led to a slight increase of anxiety‐related behaviour, though only in wild types.

We could speculate several mechanisms contributing to the neurobehavioural and cognitive improvement. It has been previously reported that upregulation of Line1 triggers an IFN‐I response in Sirt6‐knockout and normal aged mice, and that pathologies and lifespan of Sirt6 progeroid mice can be improved with NRTIs.^15^ Recent work showed a constant activation of the type 1 interferon (IFN‐I) response in DS^19^ that may contribute to brain neuropathology, as the IFN‐I response is characteristic of cellular senescence.^20^ Moreover, in DS, inflammatory biomarkers are strongly associated with intellectual disability and obesity risk.^21^, ^22^ The fact that lamivudine rescued the cognitive phenotype suggests that IFN‐I overactivation in DS^23^ may be contributed by increased retrotransposition, driven by LINE‐1 reactivation. De Cecco et al.^24^ found that IFN‐I response is overactive in human senescent cells and in aged mice, where treatment with lamivudine downregulated IFN‐I activation and age‐associated inflammation.^24^ Individuals with trisomy 21 start to age prematurely^25^ presenting early in life conditions such as AD, strongly contributed by overexpression of *APP* or *DYRK1A*. Increased copy numbers and overexpression of *APP* gene associated with sporadic AD have been linked to APP somatic gene recombination.^26^ This form of gene recombination produces internally truncated RNA sequences containing intra‐exonic junctions (IEJs), and a recent study using single nucleus RNA sequencing [8] has shown novel truncated RNAs containing IEJs and involving not only *APP* but also thousands of other genes in DS. It might thus be speculated that the observed beneficial effects of lamivudine could be due to the inhibition of the generation of one or more non‐classical variant(s) of the *APP* gene, as APP overexpression is involved in DS phenotypes.^27^

In summary, our study demonstrated for the first time that lamivudine improves cognition in a mouse model of DS, providing experimental evidence for the use of reverse transcriptase inhibitors as a new potential treatment for ameliorating cognitive impairment in DS. Further studies to assess safety and tolerability in DS patients with early stage AD are warranted.

## AUTHOR CONTRIBUTIONS

**Maria Martinez de Lagran:** Formal analysis (lead); investigation (lead); methodology (lead); writing – original draft (equal); writing – review and editing (equal). **Aleix Elizalde‐Torrent:** Methodology (supporting); writing – original draft (equal); writing – review and editing (equal). **Roger Paredes:** Conceptualization (equal); supervision (equal); writing – review and editing (equal). **Bonaventura Clotet:** Conceptualization (lead); funding acquisition (equal); supervision (equal); writing – review and editing (equal). **Mara Dierssen:** Conceptualization (equal); funding acquisition (lead); investigation (lead); project administration (lead); resources (lead); supervision (lead); writing – original draft (lead); writing – review and editing (lead).

## CONFLICT OF INTEREST

The authors declare no conflict of interest.

## Data Availability

The original contributions presented in the study are included in the article/Supplementary Material, further inquiries can be directed to the corresponding author.
